# Sulfur-Oxidizing Symbionts without Canonical Genes for Autotrophic CO_2_ Fixation

**DOI:** 10.1128/mBio.01112-19

**Published:** 2019-06-25

**Authors:** Brandon K. B. Seah, Chakkiath Paul Antony, Bruno Huettel, Jan Zarzycki, Lennart Schada von Borzyskowski, Tobias J. Erb, Angela Kouris, Manuel Kleiner, Manuel Liebeke, Nicole Dubilier, Harald R. Gruber-Vodicka

**Affiliations:** a Max Planck Institute for Marine Microbiology, Bremen, Germany; b Max Planck Genome Centre Cologne, Max Planck Institute for Plant Breeding Research, Cologne, Germany; c Max Planck Institute for Terrestrial Microbiology, Marburg, Germany; d Energy Bioengineering and Geomicrobiology Group, University of Calgary, Calgary, Alberta, Canada; e Department of Plant and Microbial Biology, North Carolina State University, Raleigh, North Carolina, USA; f MARUM, Center for Marine Environmental Sciences, University of Bremen, Bremen, Germany; Oregon State University

**Keywords:** gammaproteobacteria, chemosynthesis, ectosymbiont, lithoheterotrophy, meiofauna, protist

## Abstract

Since the discovery of symbioses between sulfur-oxidizing (thiotrophic) bacteria and invertebrates at hydrothermal vents over 40 years ago, it has been assumed that autotrophic fixation of CO_2_ by the symbionts drives these nutritional associations. In this study, we investigated “*Candidatus* Kentron,” the clade of symbionts hosted by *Kentrophoros*, a diverse genus of ciliates which are found in marine coastal sediments around the world. Despite being the main food source for their hosts, Kentron bacteria lack the key canonical genes for any of the known pathways for autotrophic carbon fixation and have a carbon stable isotope fingerprint that is unlike other thiotrophic symbionts from similar habitats. Our genomic and transcriptomic analyses instead found metabolic features consistent with growth on organic carbon, especially organic and amino acids, for which they have abundant uptake transporters. All known thiotrophic symbionts have converged on using reduced sulfur to gain energy lithotrophically, but they are diverse in their carbon sources. Some clades are obligate autotrophs, while many are mixotrophs that can supplement autotrophic carbon fixation with heterotrophic capabilities similar to those in Kentron. Here we show that Kentron bacteria are the only thiotrophic symbionts that appear to be entirely heterotrophic, unlike all other thiotrophic symbionts studied to date, which possess either the Calvin-Benson-Bassham or the reverse tricarboxylic acid cycle for autotrophy.

## Abstract (precis)

Many animals and protists depend on symbiotic sulfur-oxidizing bacteria as their main food source. These bacteria use energy from oxidizing inorganic sulfur compounds to make biomass autotrophically from CO_2_, serving as primary producers for their hosts. Here we describe a clade of nonautotrophic sulfur-oxidizing symbionts, “*Candidatus* Kentron,” associated with marine ciliates. They lack genes for known autotrophic pathways and have a carbon stable isotope fingerprint heavier than other symbionts from similar habitats. Instead, they have the potential to oxidize sulfur to fuel the uptake of organic compounds for heterotrophic growth, a metabolic mode called chemolithoheterotrophy that is not found in other symbioses. Although several symbionts have heterotrophic features to supplement primary production, in Kentron they appear to supplant it entirely.

## INTRODUCTION

Chemosynthetic symbioses between heterotrophic, eukaryotic hosts and bacteria that use the oxidation of inorganic chemicals or methane to fuel growth are common in marine environments. They occur in habitats ranging from deep-sea vents and seeps, where they are responsible for much of the primary production, to the interstitial pore water habitat in shallow-water sediments, where the hosts are often small and inconspicuous meiofauna. Among the energy sources for chemosynthesis are reduced sulfur species like sulfide and thiosulfate, and such sulfur-oxidizing (thiotrophic) symbioses have convergently evolved multiple times (1). They are commonly interpreted as nutritional symbioses where the symbionts fix CO_2_ autotrophically into biomass with the energy from sulfur oxidation and eventually serve as food for their hosts (1, 2). Indeed, several host groups have become so completely dependent on their symbionts for nutrition that they have reduced digestive systems. All sulfur-oxidizing symbioses investigated thus far possess a primary thiotrophic symbiont with genes of either the Calvin-Benson-Bassham (CBB) (3–10) or reverse tricarboxylic acid (rTCA) (11, 12) cycle for CO_2_ fixation, and the different pathways may relate to different ecological niches occupied by the symbioses (13). The symbionts of the vestimentiferan tubeworms are additionally able to encode both the CBB and rTCA cycles, which may be active under different environmental conditions (14–16). Beyond sulfur oxidation and carbon fixation, several thiotrophic symbionts have additional metabolic capabilities such as the uptake of organic carbon (17), the use of carbon monoxide (18) and hydrogen (19) as energy sources, and the ability to fix inorganic nitrogen (4, 5).

The thiotrophic ectosymbionts of the ciliate genus *Kentrophoros* constitute a distinct clade of *Gammaproteobacteria* named “*Candidatus* Kentron” (here Kentron) (20). Kentron has previously been shown with radioisotope labeling experiments to oxidize sulfide and fix CO_2_ (21) and to be consumed and digested by its hosts (21, 22). Unlike most ciliates, which consume their food at a specific location on the cell that bears feeding structures composed of specialized cilia, *Kentrophoros* has only vestiges of such cilia and instead directly engulfs its symbionts along the entire cell body (23), suggesting that Kentron bacteria are its main food source.

Given that all previous studies of thiotrophic symbionts, including Kentron, have characterized them as autotrophic, we expected that the pathways of energy and carbon metabolism used by Kentron would resemble those in other thiotrophic bacteria involved in nutritional symbioses. In this study, we used metagenomic and transcriptomic analyses of single host individuals to show that the Kentron clade lacks the canonical pathways of autotrophic CO_2_ fixation. Based on a metabolic reconstruction of the core genome from 11 Kentron phylotypes collected from three different sites, and results from direct protein-stable isotope fingerprinting, we propose that it is a lithoheterotrophic nutritional symbiont, relying on assimilation of organic substrates rather than fixation of inorganic carbon to feed its hosts.

## RESULTS

### Symbiont genome assemblies have high coverage and completeness and represent 11 phylotypes.

**FIG 1:**
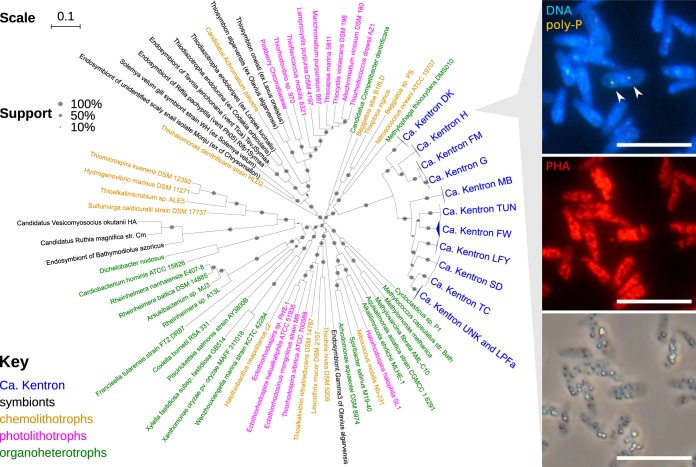
Maximum-likelihood phylogeny of Kentron and basal *Gammaproteobacteria* from concatenated alignment of 30 conserved protein-encoding marker genes. Support values, SH-like approximate likelihood ratio test (aLRT). Branch lengths, substitutions per site. (Inset) Kentron sp. H fluorescence with DAPI stain for polyphosphate (top, arrowheads) and Nile Red stain for PHA (middle) and in bright field showing refractile sulfur globules (bottom). Bars, 10 μm.

Genomes of Kentron symbionts were binned from 34 metagenome assemblies, each corresponding to a single *Kentrophoros* host ciliate individual. The genomes represented 11 symbiont phylotypes from 12 host morphospecies (Fig. 1), collected from the Mediterranean, Caribbean, and Baltic Seas (see data posted at https://doi.org/10.5281/zenodo.2575755). Most symbiont phylotypes were specific to the host morphospecies, except for one symbiont associated with two hosts (*Kentrophoros* sp. UNK and *Kentrophoros* sp. LPFa). Symbiont genome assemblies were between 3.31 and 5.02 Mbp (median, 3.91 Mbp) long but were relatively fragmented (*N*_50_: 3.52 to 37.5 kbp; median, 21.4 kbp). Genome sizes and assembly fragmentation appeared to be species/phylotype dependent (see Fig. S1A and B in the supplemental material). Nonetheless, they were relatively complete (91.4 to 94.9%; median, 93.8%) and had low contamination (0.75 to 3.56%; median, 1.87%) (see data posted at https://doi.org/10.5281/zenodo.2575767). The core genomic diversity in the clade was well sampled: 1,019 protein-encoding gene orthologs were found in all 34 genomes, and the core genome accumulation curve reached a plateau (Text S1; Fig. S1C). Kentron genome sizes were relatively large for thiotrophic symbionts and were comparable to values for “*Candidatus* Thiodiazotropha” spp. (4.5 Mbp) and the Gamma3 symbiont of Olavius algarvensis (4.6 Mbp).

10.1128/mBio.01112-19.1 TEXT S1 Phylogenetics. Download Text S1, PDF file, 0.1 MB. Copyright © 2019 Seah et al. 2019 Seah et al. This content is distributed under the terms of the Creative Commons Attribution 4.0 International license.

10.1128/mBio.01112-19.4 FIG S1 Summary statistics for Kentron genome assemblies. (A) Genome completeness per species, as assessed with gammaproteobacterial conserved marker genes by CheckM. (B*)* Number of contigs versus total length (Mbp) of Kentron genome bins; plot symbol areas scaled by *N*_50_ (kbp). Larger genomes are usually more fragmented (more contigs, lower *N*_50_), with the exception of Kentron sp. DK. (C) Accumulation curves for pan- and core genomes (protein-encoding genes only) of the Kentron clade, with uncertainty estimated by resampling (200 times, with replacement). “Singleton” genes were not included in an ortholog cluster. Download FIG S1, EPS file, 0.1 MB. Copyright © 2019 Seah et al. 2019 Seah et al. This content is distributed under the terms of the Creative Commons Attribution 4.0 International license.

### Genes for key enzymes in known autotrophic pathways are absent.

Unlike other investigated thiotrophic symbionts, genes for ribulose-1,5-bisphosphate carboxylase/oxygenase (RuBisCO) and other key enzymes in the six canonical autotrophic CO_2_ fixation pathways (see data posted at https://doi.org/10.5281/zenodo.2575773) were not predicted in Kentron genomes by standard annotation pipelines. Kentron sp. H has a gene for group IV RuBisCO (Fig. S2), also known as RuBisCO-like protein (RLP), which is not known to play a role in carbon fixation but participates in a variety of other pathways such as thiosulfate metabolism (24).

10.1128/mBio.01112-19.5 FIG S2 Maximum-likelihood phylogeny of RuBisCO proteins, including RuBisCO-like protein from Kentron phylotype H (red arrow). Download FIG S2, EPS file, 0.5 MB. Copyright © 2019 Seah et al. 2019 Seah et al. This content is distributed under the terms of the Creative Commons Attribution 4.0 International license.

**FIG 2:**
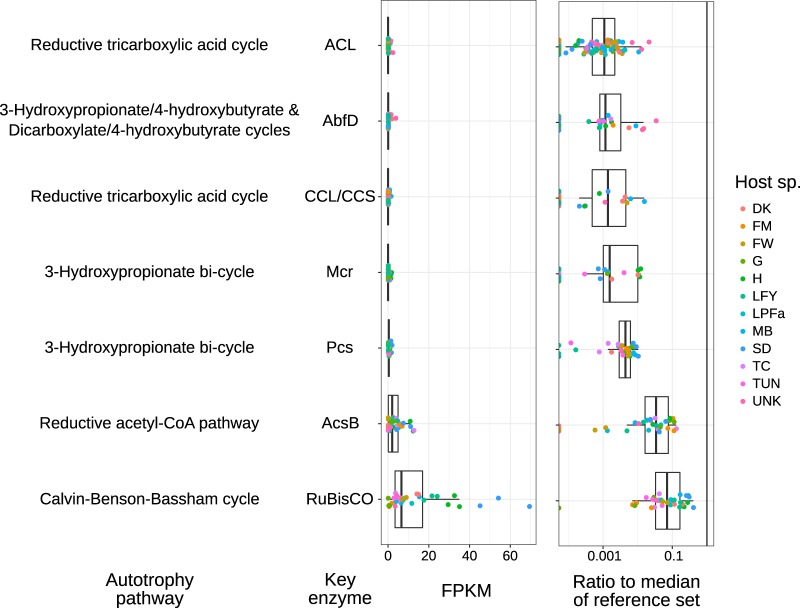
Read coverage (individual values and box plots) in *Kentrophoros* metagenomes for key enzymes of autotrophic CO_2_ fixation pathways, expressed as FPKM values (left) and as a fraction of the median coverage of a reference set of proteins that are expected to be present in all Kentron species (right) (see data posted at https://doi.org/10.5281/zenodo.2575773). Unassembled reads were used for alignment, to rule out the possibility of not detecting hits because of misassembly. Each point represents a separate metagenome library, colored by *Kentrophoros* host morphospecies. The box midline represents the median, hinges represent the interquartile range (IQR), and whiskers are data within 1.5× IQR of hinges. Abbreviations: ACL, ATP citrate lyase; AbfD, 4-hydroxybutanoyl-CoA dehydratase; CCL/CCS, citryl-CoA lyase/citryl-CoA synthase; Mcr, malonyl-CoA reductase; Pcs, propionyl-CoA synthase; AcsB, CO-methylating acetyl-CoA synthase; RuBisCO, ribulose-1,6-bisphosphate carboxylase/oxygenase.

To rule out the possibility of misannotation, incomplete genome binning, or problems with genome assembly, we aligned raw, unassembled reads from *Kentrophoros* metagenome libraries to the curated Swiss-Prot database of protein sequences. Key autotrophy proteins had coverage values (median of 0.00, maximum of 69.3 fragments per kilobase per million [FPKM]) that were always lower than the median coverage of reference proteins from the TCA and partial 3-hydroxypropionate (3HPB) pathways (Fig. 2). In 89% of cases, the coverage was at least 50-fold lower than the reference median, and if not, the majority of reads could be attributed either to other microbial genome bins in the metagenome (mostly RuBisCO or AcsB) or to the RuBisCO-like protein in Kentron H (Fig. S3). Metatranscriptomes of two phylotypes (H and SD) were also screened with the same pipeline, and key autotrophy proteins again had coverages that were always below the median of the reference set (median, 0.00; maximum, 1.62 FPKM) (Fig. S4). We conclude that canonical autotrophy genes were indeed absent from Kentron genomes and not merely misassembled or mispredicted.

10.1128/mBio.01112-19.6 FIG S3 Selected results of remapping reads with hits to canonical autotrophy enzymes to the metagenomic assemblies, for AcsB versus *Kentrophoros* sp. TC (left) and RuBisCO versus *Kentrophoros* sp. H (right). Metagenome assemblies are depicted as coverage-GC% plots, where each gray circle represents a contig, with its size scaled to the contig length. Contigs with remapping hits have solid outlines, with numbers of remapping reads indicated when >10. The majority of reads with alignments to canonical autotrophy enzymes map to scaffolds outside the Kentron symbiont genome bin (circled in blue) and could be attributed to other bacteria in the metagenomes, except for RuBisCO in Kentron sp. H, due to the RuBisCO-like protein encoded in that phylotype. Download FIG S3, TIF file, 0.3 MB. Copyright © 2019 Seah et al. 2019 Seah et al. This content is distributed under the terms of the Creative Commons Attribution 4.0 International license.

**FIG 3:**
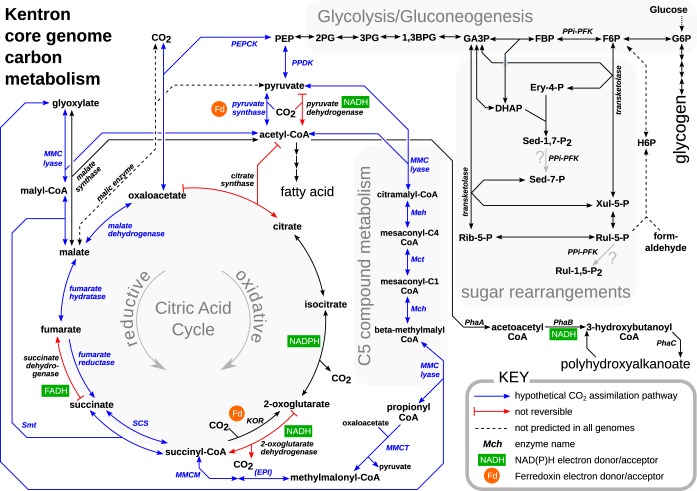
Schematic reconstruction of carbon and central metabolism of Kentron clade, focusing on pathways discussed in the text. Compound name abbreviations: 1,3BPG, 1,3-bisphosphoglycerate; 2PG, 2-phosphoglycerate; 3PG, 3-phosphoglycerate; DHAP, dihydroxyacetone phosphate; Ery-4-P, erythrose-4-phosphate; F6P, fructose-6-phosphate; FBP, fructose-1,6-bisphosphate; G6P, glucose-6-phosphate; GA3P, glyceraldehyde-3-phosphate; H6P, hexose-6-phosphate; PEP, phosphoenolpyruvate; Rib-5-P, ribose-5-phosphate; Rul-1,5-P_2_, ribulose-1,5-bisphosphate; Rul-5-P, ribulose-5-phosphate; Sed-1,7-P_2_, sedoheptulose-1,7-bisphosphate; Sed-7-P, sedoheptulose-7-phosphate; Xul-5-P, xylulose-5-phosphate. Enzyme name abbreviations: EPI, methylmalonyl-CoA epimerase; KOR, alpha-ketoglutarate oxidoreductase; Mch, mesaconyl-C_1_-CoA hydratase; Mct, mesaconyl-CoA C_1_-C_4_ CoA transferase; Meh, mesaconyl-C_4_-CoA hydratase; MMC lyase, (*S*)-malyl-CoA/beta-methylmalyl-CoA/(*S*)-citramalyl-CoA lyase; MMCM, methylmalonyl-CoA mutase; MMCT, methylmalonyl-CoA carboxytransferase; PEPCK, phosphoenolpyruvate carboxykinase; PPDK, pyruvate phosphate dikinase; PPi-PFK, pyrophosphate-dependent phosphofructokinase; Smt, succinyl-CoA:(*S*)-malate-CoA transferase.

10.1128/mBio.01112-19.7 FIG S4 Read coverage in *Kentrophoros* metatranscriptomes for key enzymes of autotrophic CO_2_ fixation pathways, expressed as FPKM values (left) and as a fraction of the median coverage of a reference set of proteins (right). Each point represents a separate metatranscriptome library, colored by *Kentrophoros* host morphospecies. Abbreviations as in Fig. 3. Download FIG S4, EPS file, 0.1 MB. Copyright © 2019 Seah et al. 2019 Seah et al. This content is distributed under the terms of the Creative Commons Attribution 4.0 International license.

### Evidence for lithoheterotrophic metabolism in Kentron.

Kentron genome annotations suggested a lithoheterotrophic metabolism, in which energy is produced by oxidation of reduced sulfur and carbon is assimilated in the form of organic compounds (Fig. 3).

**FIG 4:**
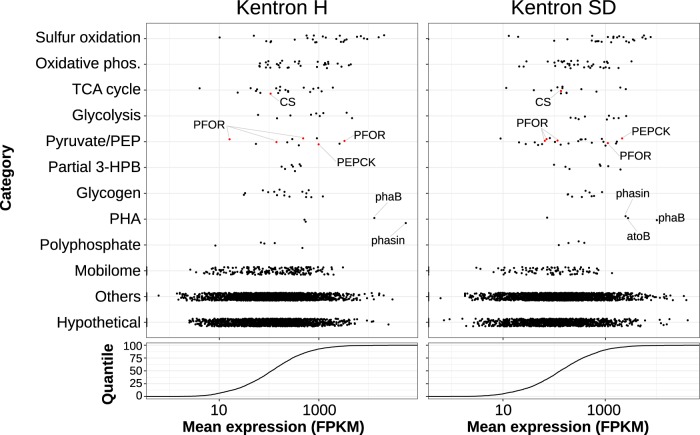
Expression of genes in selected functional categories, for two Kentron phylotypes (mean for 3 samples per phylotype, in FPKM). Cumulative distribution curves for expression per gene are shown below each dot plot. Selected genes discussed in the text are labeled; carboxylation reactions involving pyruvate or phosphoenolpyruvate (PEP) are marked in red. Abbreviations: atoB, acetyl-CoA acetyltransferase; CS, citrate synthase; PFOR, pyruvate-ferredoxin oxidoreductase (=pyruvate synthase); PEPCK, phosphoenolpyruvate carboxykinase; phaB, acetoacetyl-CoA reductase.

**(i) Electron donors and energetics.** Kentron genomes encoded a hybrid Sox-reverse Dsr pathway, similar to other symbiotic and free-living thiotrophs (e.g., Allochromatium vinosum), which would allow the oxidation of thiosulfate, elemental sulfur, and sulfide as energy sources (25, 26). They had a complete electron transport chain for oxidative phosphorylation and an F_o_F_1_-type ATP synthase. The only terminal oxygen reductase predicted was *cbb*_3_-type cytochrome *c* oxidase (complex IV), which has a high oxygen affinity and is typically expressed under microoxic conditions (27, 28). In the two Kentron phylotypes for which expression profiles were available, this set of functions was among the most highly expressed genes (Fig. 4).

Four Kentron phylotypes (H, SD, FW, and G) encoded anaerobic-type Ni-dependent CO dehydrogenase precursors, adjacent to CO dehydrogenase Fe-S subunits (in FW and SD) or a CO dehydrogenase maturation factor (in G). In addition, H_2_ may serve as an electron donor for Kentron TC, TUN, G, and FW (one genome), which contained genes related to the oxidative-type [Ni-Fe] hydrogenase Mvh (A and G subunits), as well as auxiliary proteins for hydrogenase maturation and Ni incorporation, although they did not all occur in a single gene cluster. Both CO and H_2_ are known to be potential electron donors for symbiotic thiotrophs and have been measured in their habitat in Sant’ Andrea, Elba (18), where one of these *Kentrophoros* phylotypes (H) was collected.

Oxidoreductases for anaerobic respiration were not predicted, except for subunits NapA and -B of periplasmic nitrate reductase (in 28 and 25 genomes, respectively). Other oxidoreductases for complete denitrification or the dissimilatory nitrate reduction to ammonia pathway were absent. Some organisms can respire nitrate with only Nap, but because Nap has other potential functions, it is not possible to predict if Kentron can respire nitrate from the presence of Nap alone (29). Na^+^-translocating ferredoxin:NAD^+^ (Rnf) and NADH:ubiquinone (Nqr) oxidoreductases, which can couple reducing equivalents to the Na^+^ membrane potential, were also predicted.

**(ii) Uptake transporters for organic substrates.** Genes encoding uptake transporters for organic substrates were abundant in Kentron genomes and were also expressed in the transcriptomes (Fig. S5; see also File 2 posted at https://doi.org/10.5281/zenodo.2555833). An average of 54.1 such genes were predicted per genome (representing 18.1% of all genes with Transporter Classification Database [TCDB] hits), of which more than half had transmembrane (TM) domains (mean, 30.5 per genome). The families with the highest mean counts per genome were the ATP-binding cassette (ABC) superfamily (33.9 total, 16.4 transmembrane, counting only uptake-related subfamilies), tripartite ATP-independent periplasmic transporter (TRAP-T) family (7.2 total, 5.1 TM), and the solute:sodium symporter (SSS) family (1.6 total, 1.3 TM). Three other families—concentrative nucleoside transporter (CNT), dicarboxylate/amino acid cation symporter (DAACS), and neurotransmitter/sodium symporter (NSS)—were represented by a single gene in all Kentron genomes. Most of these families are known to target organic acids, amino acids, or small peptides. In comparison, sugar uptake transporter families were less numerous and present in only a subset of genomes (e.g., ABC subfamilies CUT1 and CUT2) or not predicted in Kentron at all (e.g., phosphotransferase system family).

10.1128/mBio.01112-19.8 FIG S5 Uptake-related transporter families found in all Kentron genomes. (a) Number of open reading frames per genome with matches to Transporter Classification families (Blastp E value <10^−5^, >30% amino acid identity, >70% query coverage). (b) Mean expression (FPKM) in three transcriptomes of Kentron H. (c) Mean expression (FPKM) in three transcriptomes of Kentron SD. Download FIG S5, TIF file, 0.3 MB. Copyright © 2019 Seah et al. 2019 Seah et al. This content is distributed under the terms of the Creative Commons Attribution 4.0 International license.

**FIG 5:**
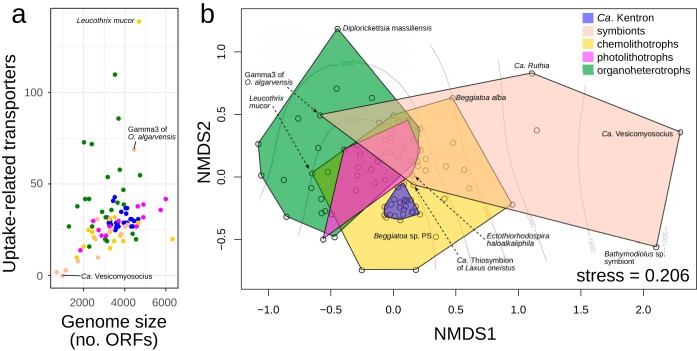
Comparison of organic substrate transporters in genomes of Kentron and other basal *Gammaproteobacteria*, named in Fig. 1. (a) Counts of uptake-related transporters (transmembrane only) versus genome size (expressed in numbers of open reading frames). (b) Two-dimensional ordination plot (nonmetric multidimensional scaling) of genomes based on counts of uptake-related Transporter Classification families and subfamilies per genome. Bray-Curtis distance metric; stress = 0.206. Contour lines indicate approximate genome size. Colors in both plots have the same legend and represent types of metabolism.

The number of organic uptake transporters in Kentron was high compared to other symbiotic thiotrophs, which had counts ranging from 2 (0 TM) in “*Candidatus* Vesicomyosocius okutanii” to 134 (69 TM) in the Gamma3 symbiont of *Olavius algarvensis*. However, larger genomes tend to have more transporters, and Kentron genomes were also relatively large (Fig. 5a). We therefore compared the content of organic-uptake-related TCDB family members per genome between Kentron and other basal gammaproteobacteria by nonmetric multidimensional scaling. Kentron overlapped the known range of variation for both phototrophs and chemolithotrophs (both free-living and symbiotic). Among heterotrophs, it was most distant from pathogens like *Diplorickettsia*. Among thiotrophic symbionts, it was most similar to “*Candidatus* Thiosymbion” but most distant from the symbionts of deep-sea bivalves (which have few uptake transporters) (Fig. 5b).

**(iii) Heterotrophic carbon metabolism.** Kentron genomes encoded both glycolysis (Embden-Meyerhoff-Parnas pathway) and the oxidative tricarboxylic acid (TCA) cycle. The canonically irreversible reactions of glycolysis, pyruvate kinase and phosphofructokinase, were replaced in Kentron by reversible, pyrophosphate-dependent alternatives pyruvate phosphate dikinase and PP_i_-dependent phosphofructokinase (PP_i_-PFK), respectively. These alternatives have been found in other thiotrophic symbioses, where they may make the CBB cycle more energy efficient (30).

Genes for pyruvate dehydrogenase and the complete oxidative TCA cycle were present, including 2-oxoglutarate dehydrogenase, which is often missing in obligate autotrophs (31). The reductive equivalents for the key steps of the oxidative TCA cycle were also present, namely, ferredoxin-dependent pyruvate synthase (PFOR), ferredoxin-dependent 2-oxoglutarate synthase, and fumarate reductase. However, because neither ATP citrate lyase (ACL) nor citryl coenzyme A (CoA) lyase/citryl-CoA synthase (CCL/CCS) was predicted, a canonical autotrophic reductive TCA cycle was not predicted.

Heterotrophic carboxylases also had relatively high expression levels. PFOR was present in multiple copies per genome, of which the highest expressed were at the 98.4 and 93.0 percentiles in Kentron H and SD, respectively (Fig. 4). GDP-dependent phosphoenolpyruvate (PEP) carboxykinase (PEPCK), which can replenish oxaloacetate anaplerotically, was also highly expressed (93.0 and 96.7 percentiles) (Fig. 4). Unlike PEP carboxylase, which was not predicted, PEPCK catalyzes a reversible reaction.

Pathways that enable growth solely on acetate as both energy and carbon source were either incomplete (glyoxylate shunt) or not predicted (ethylmalonyl-CoA pathway and methylaspartate cycle).

**(iv) Partial 3-hydroxypropionate bi-cycle.** Genes encoding most enzymes of the 3-hydroxypropionate bi-cycle (3HPB), which is the autotrophic pathway used by members of the distant bacterial phylum *Chloroflexi*, were predicted in Kentron. These genes had expression levels in the 64.1 to 83.0 and 48.2 to 96.4 percentile ranges for Kentron H and SD, respectively (Fig. 4). The key enzymes malonyl-CoA reductase and propionyl-CoA synthase were absent; hence, the bi-cycle was not closed and would not function autotrophically. However, the remainder of the pathway could function in the assimilation of organic substrates (e.g., acetate and succinate) or to connect metabolite pools (acetyl-CoA, propionyl-CoA, pyruvate, and glyoxylate) (32), as previously proposed for *Chloroflexus* (33) and the “*Ca*. Thiosymbion” symbionts of gutless oligochaetes (3).

These enzymes are unusual because their genes are uncommon and have a disjunct phylogenetic distribution: *Chloroflexi*, at least four clades in *Gammaproteobacteria* (Kentron, “*Ca*. Thiosymbion,” “*Candidatus* Competibacter,” “Pink Berry” *Chromatiaceae*), a symbiotic *Alphaproteobacteria* member (“*Candidatus* Riegeria”) (34), and *Betaproteobacteria* (“*Candidatus* Accumulibacter”). While they were previously thought to have been horizontally transferred from *Chloroflexi* to the other groups (33), gene phylogenies show that the *Chloroflexi* probably also gained the 3HPB by horizontal transfer (35), which was supported by our analysis with Kentron homologs (Fig. S6).

10.1128/mBio.01112-19.9 FIG S6 Phylogenetic trees of genes of 3-hydroxypropionate bi-cycle found in Kentron, with homologs from other bacteria. Abbreviations: Mch, mesaconyl-C_1_-CoA hydratase; Mcl, malyl-CoA/beta-methylmalyl-CoA/citramalyl-CoA (MMC) lyase; Mct, mesaconyl-CoA C_1_-C_4_ CoA transferase; Meh, mesaconyl-C_4_-CoA hydratase. Download FIG S6, EPS file, 0.5 MB. Copyright © 2019 Seah et al. 2019 Seah et al. This content is distributed under the terms of the Creative Commons Attribution 4.0 International license.

**(v) Storage compounds.** In addition to elemental sulfur, Kentron also has the potential to store and mobilize carbon (as polyhydroxyalkanoates [PHAs] and starch/glycogen) and phosphorus (as polyphosphate). PHA was detected with the lipophilic fluorescent dye Nile Red, and polyphosphate was detected by metachromatic yellow fluorescence emission of DAPI (Fig. 1, inset). Genes related to PHA synthesis were among the most highly expressed, namely, those encoding phasin, a protein associated with the surface of PHA granules, and putative acetoacetyl-CoA reductase (*phaB*) (Fig. 4). Trehalose was detected in *Kentrophoros* sp. H but is probably produced and accumulated by the host ciliate rather than the symbionts (Text S2).

10.1128/mBio.01112-19.2 TEXT S2 Metabolomics. Download Text S2, PDF file, 0.1 MB. Copyright © 2019 Seah et al. 2019 Seah et al. This content is distributed under the terms of the Creative Commons Attribution 4.0 International license.

### Proteomics-based carbon SIF of Kentron.

**FIG 6:**
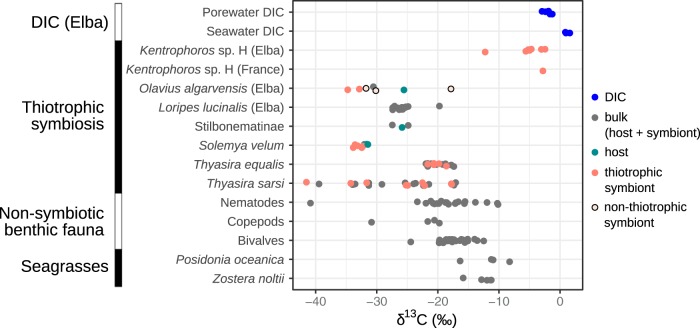
Carbon stable isotope δ-^13^C composition values in *Kentrophoros* sp. H (this study) and published values for other shallow-water thiotrophic symbioses (5, 37–40), nonsymbiotic benthic animals (5, 60, 61), and two Mediterranean seagrass species (60–63), compared with dissolved inorganic carbon (DIC) from pore water and seawater at Elba (this study). Values for *Kentrophoros* and *Olavius algarvensis* (except the “bulk” value) are from direct protein-SIF, and others are from isotope ratio mass spectrometry (IRMS). Values for symbiont-bearing tissue (e.g., gills) are also included under “symbiont.”

Measuring the natural abundance ratio of carbon stable isotopes ^13^C/^12^C, also known as the stable isotope fingerprint (SIF), is a challenge in *Kentrophoros* because of its small biomass (∼10^6^ symbionts and ∼10 μg [wet weight] per ciliate in the largest species). Sensitive applications of isotope ratio mass spectrometry (IRMS) require at least ∼10^7^ bacterial cells (36), and compound-specific IRMS for signatures of specific pathways like ^13^C enrichment in fatty acids in the rTCA cycle (11) requires considerably more. We therefore used a newly developed metaproteomics method that could distinguish the SIF of the symbiont from other biomass in the sample (37). The protein-based carbon SIF for Kentron sp. H from Elba and France ranged from −12.3 to −2.5‰ (*n* = 8), expressed as δ-^13^C values (Fig. 6; see also data posted at https://doi.org/10.5281/zenodo.2575800). In comparison, other shallow-water thiotrophic symbioses, from the same locality and elsewhere, possess the CBB cycle and have δ-^13^C values of <−17‰ and generally in the range of −30 to −20‰ (5, 37–40). The δ-^13^C of dissolved inorganic carbon (DIC) in pore water from Elba was between −2.99 and −1.32‰ (Fig. 6; see also data posted at https://doi.org/10.5281/zenodo.2575803).

## DISCUSSION

In this study, we have shown that the Kentron symbionts of *Kentrophoros* ciliates are unique among thiotrophic symbionts because they do not encode canonical pathways for autotrophic carbon fixation but have a variety of heterotrophic features. Their carbon stable isotope fingerprints are also substantially heavier than other thiotrophic symbioses from similar habitats. We therefore propose that Kentron bacteria are chemolithoheterotrophs (41), oxidizing inorganic compounds (in this case, reduced sulfur species) to provide energy for assimilating organic carbon as the main carbon source for growth.

### Role of heterotrophic CO_2_ fixation.

Our results conflict with a previous study that concluded that Kentron is an autotroph, based on uptake of ^14^C-labeled bicarbonate, at a maximum rate of 0.117 bacterial cell carbon h^−1^ under oxic conditions (21). The CO_2_ fixation rates were lower than expected, but this was attributed to the artificial experimental conditions. To rule out the possibility that only some species are autotrophs, we collected *Kentrophoros* matching the described morphology from the same site (Nivå Bay, Denmark), but their symbionts (phylotype DK) lacked canonical autotrophic pathways like other Kentron phylotypes.

However, the ability to fix CO_2_ alone is insufficient evidence for autotrophy, which is defined as the ability to grow with inorganic carbon as the sole or major carbon source (42). Heterotrophs can also fix CO_2_ to some extent, e.g., via anaplerotic reactions in the oxidative TCA cycle (43). Such heterotrophic fixation can account theoretically for up to a third of total cell carbon (44) and has been measured at 10% or more in some bacteria (45, 46). The previous study (21) measured assimilation only of CO_2_, but not of total carbon, nor the growth rate of the cells. Instead, the authors assumed that all CO_2_ fixed was autotrophic and argued that the resulting calculated growth rate was low but plausible.

We reinterpret their results as heterotrophic CO_2_ fixation during the mobilization of the storage polymer polyhydroxyalkanoate (PHA) to biomass, which involves the heterotrophic carboxylation of acetyl-CoA to pyruvate by ferredoxin-dependent pyruvate synthase (PFOR). This is plausible because their experiments were performed with freshly collected cells that had visible cellular inclusions, and Kentron uses PHA as a carbon store (Fig. 1 and 3). In our data, genes for PFOR and another heterotrophic carboxylase, PEP carboxykinase (PEPCK), were highly expressed, suggesting that these enzymes are indeed present and functionally important (Fig. 4).

Furthermore, the previous study (21) also observed that adding sulfide or thiosulfate decreased the CO_2_ fixation rates, although sufficient electron acceptor was available. A lithoautotroph should instead fix more CO_2_ under such conditions, as observed in other thioautotrophic symbioses (47). This decrease is, however, consistent with our interpretation of heterotrophic CO_2_ fixation during PHA mobilization, because PHA conversion to biomass is a net oxidation reaction (see Text S3 in the supplemental material), which would not be favored under reducing conditions.

10.1128/mBio.01112-19.3 TEXT S3 Metabolic reconstruction details. Download Text S3, PDF file, 0.1 MB. Copyright © 2019 Seah et al. 2019 Seah et al. This content is distributed under the terms of the Creative Commons Attribution 4.0 International license.

### Could Kentron use a novel autotrophic CO_2_ fixation pathway?

Different autotrophic carbon fixation pathways each have characteristic degrees of isotope fractionation discriminating against the heavier isotope ^13^C, resulting in biomass that is relatively depleted in ^13^C (i.e., more negative δ-^13^C values) (48). Kentron bacteria were more enriched in ^13^C than other shallow-water thiotrophic symbioses and showed only a modest ^13^C depletion relative to DIC from the same site (Fig. 6), which ruled out the possibility that they use a pathway with strong isotope fractionation (ε), such as the CBB cycle (ε = 10 to 22‰) or the reductive acetyl-CoA pathway (ε = 15 to 36‰) (49). Other pathways such as the reverse TCA cycle (ε = 4 to 13‰) or 3-hydroxypropionate bicycle (ε ≈ 0‰) may still fall in this range, but given that the key genes for these pathways were not detected, this possibility would require the postulation of hitherto-unknown enzymes.

In two different thermophilic bacteria, the oxidative TCA cycle has recently been found to function in the autotrophic direction without using ACL or CCL/CCS, but instead by reversing the citrate synthase reaction. Such a “reversed oxidative TCA” (roTCA) cycle would not be distinguishable from the oxidative TCA by genome sequences alone (50, 51). However, both roTCA bacteria require anoxic conditions with hydrogen as the energy source for autotrophic growth. They are also facultative autotrophs and switch to heterotrophic growth when suitable substrates like acetate are available. Citrate synthase is also highly expressed in the roTCA, whereas in Kentron the gene has only moderate expression (Fig. 4, 51.2 and 56.3 percentiles in Kentron H and SD, respectively). For these reasons, it is unlikely that a microaerophilic sulfur oxidizer like Kentron uses the roTCA for autotrophic growth.

Alternatively, it is possible to combine several reactions predicted in Kentron, including elements of the partial 3HPB, into a hypothetical autotrophic CO_2_ fixation pathway, without proposing any novel enzymes or biochemical reactions, with PFOR and PEPCK as carboxylases (Fig. 3; Text S3). Although it is stoichiometrically and energetically feasible, it is unlikely that this is a truly functional pathway, as the same enzymes are predicted in other bacteria, including “*Ca*. Thiosymbion,” a thiotrophic symbiont that has clear genetic (3) and isotopic (37) signatures of autotrophic CO_2_ fixation with the Calvin cycle (Text S3).

### The autotrophy-heterotrophy spectrum in thiotrophic symbiosis.

Thiotrophic symbioses are most commonly found in nutrient-limited environments, and their symbionts are assumed to provide the hosts with nutrition through the autotrophic fixation of CO_2_. Indeed, the symbionts of deep-sea bivalves *Bathymodiolus* and *Calyptogena* show characteristic features of obligate autotrophy in their genomes, namely, an incomplete TCA cycle and the lack of organic uptake transporters (Table 1) (19, 52–54). This appears to be the exception, however, as other symbiont clades possess heterotrophic features to various degrees (Table 1). Some features, e.g., glycolysis, are involved in the mobilization of storage compounds, but abundant presence and expression of organic uptake transporters, as we observed in Kentron in this study, are a clearer marker of heterotrophic assimilation (55). Mixotrophic potential in other symbionts has been variously suggested to be a strategy to cope with carbon limitation by recycling host waste, as a nutritional supplement to autotrophy, or to be retained for a hypothetical free-living stage of the symbiont life cycle (3, 7, 30). Thus, we propose a spectrum among thiotrophic symbionts between obligate autotrophs and heterotrophic Kentron, with various degrees of mixotrophy in between.

**TABLE 1:** Comparison of metabolic features predicted in thiotrophic symbiont genomes^*a*^

Feature	Host habitat and organism						
	Hydrothermal vents and seeps			Shallow water sediment interstitial			
	Bathy symbiont	*Ruthia*	*Endoriftia*	*Thiodiazotropha*	*Solemya* symbiont	Thiosymbion	Kentron
Autotrophy							
CBB cycle (RuBisCO, phosphoribulokinase)	+	+	+	+	+	+	
PP_i_-phosphofructokinase	+	+	+	+	+	+	+
rTCA (citrate cleavage)			+				
Diazotrophy, nitrogenase				+		(+)	
TCA cycle							
Oxidative TCA			+	+	+	+	+
Kor and Frd (reductive TCA)			+	+		+	+
Glyoxylate shunt				+	+	+	
Central metabolism							
Pyruvate phosphate dikinase			+	+	+	+	+
PEP synthase			+	+	+	+	
Pyruvate synthase (PFOR)			+	+	+	+	+
Pyruvate carboxylase				+		+	
PEP carboxylase				+	+		
PEP carboxykinase (GTP)				+		+	+
Malic enzyme	+	+	+	+	+	+	(+)
C_5_ reactions of p3HPB						+	+
Energy							
Rnf transporter	+	+	+	+	+	+	+
V-type ATPase			+	+	+		
Cyt c oxidase cbb3 type	+	+	+	+	+	+	+
Cyt c oxidase *aa*_3_ type	+	+		+	+	+	
Storage compounds							
Glycogen			+	+	+	+	+
Polyhydroxyalkanoates				+	+	+	+
Polyphosphate synthesis				+	+	+	+

a Symbols: +, present; (+), not in all genomes. Abbreviations: Bathy, *Bathymodiolus*; CBB, Calvin-Benson-Bassham cycle; Cyt c, cytochrome *c*; Frd, fumarate reductase; Kor, 2-oxoglutarate:ferredoxin oxidoreductase; PEP, phosphoenolpyruvate; PP_i_, pyrophosphate; rTCA, reverse TCA cycle.

Lithoheterotrophy is not uncommon for free-living thiotrophs and appears to be more prevalent among those that have the Sox pathway (i.e., thiosulfate oxidizers) than those with the rDsr/Sox pathway (i.e., thiotrophs that can store and oxidize elemental sulfur) (Text S3). Moreover, some free-living thiotrophs that possess the CBB cycle may nonetheless grow only when supplied with organic substrates, e.g., freshwater *Beggiatoa* (56). Functional heterotrophy may therefore be underestimated as it is not necessarily apparent from genomic predictions.

Host biology constrains the feasibility of autotrophy for a thiotrophic symbiont. To meet nutritional requirements by chemoautotrophy alone, the host must provide high O_2_ flux to its symbionts, beyond what it requires itself (57). This is metabolically demanding, and it is telling that the bathymodioline and vesicomyid bivalves, whose symbionts have the most autotrophic features, are relatively large animals with intracellular symbionts that are located in their gill tissues, which can better maintain ventilation and homeostasis than smaller hosts that have extracellular symbionts. Specialization for high autotrophic production rates is also seen in the preconcentration of CO_2_ by the bivalve Bathymodiolus azoricus for its symbionts and in its thiotrophic symbiont’s metabolic dependence on the animal to replenish TCA cycle intermediates (58).

Meiofaunal hosts like *Kentrophoros* and stilbonematine nematodes, in contrast, are much smaller, cannot span substrate gradients, and must be able to tolerate fluctuating anoxia. Their smaller sizes (100-μm to 1-mm range) also mean that they are less constrained by diffusion limitation in nutrient uptake. Given that shallow-water coastal environments also receive more organic input, for example, from land or from seagrass beds, than deep-sea hydrothermal environments, it is not surprising that the shallow-water meiofaunal symbioses have more heterotrophic features than the deep-sea ones (Table 1).

### Ecophysiological model of the *Kentrophoros* symbiosis.

Based on our results and previous descriptions of morphology and behavior in *Kentrophoros* and other symbioses, we propose the following model for the ecophysiology of this symbiosis.

*Kentrophoros* fuels its growth by the phagocytosis and digestion of its symbionts (22). There has to be a net input of energy and organic carbon from environmental sources for the overall growth of the host-symbiont system, and heterotrophic carboxylation may also be a substantial carbon source. To give its symbionts access to these substrates, *Kentrophoros* likely shuttles between oxic and anoxic zones in marine sediment, like other motile, sediment-dwelling hosts with thiotrophic symbionts (38). In anoxic sediment, both the predicted energy and carbon sources, namely, sulfide and organic acids, are produced by microbial activity (59). Many organic acids, such as acetate and succinate, are more oxidized than average biomass (see data posted at https://doi.org/10.5281/zenodo.2575777), and the electron donor to assimilate them would be sulfide. However, if this were to occur under anoxic conditions, sulfide would not be completely oxidized to sulfate. Complete oxidation of sulfide to sulfate requires oxygen, so elemental sulfur would be the intermediate, partly oxidized form accumulated when Kentron assimilates organic acids. The synthesis of PHA from small organic acids like acetate can also function as both an additional electron sink for sulfide oxidation and a carbon store. Hydrolysis of polyphosphate and mobilization of glycogen are also potential sources of energy in the absence of oxygen.

Under oxic conditions, elemental sulfur can be further oxidized to sulfate for energy, and PHA can be mobilized for biosynthesis. Glycogen and polyphosphate reserves can also be regenerated. The various storage inclusions in Kentron, namely, elemental sulfur, PHA, glycogen, and polyphosphate, hence represent pools of energy, reducing equivalents, and carbon that function as metabolic buffers for the symbiont living in a fluctuating environment.

The symbionts may also bring a syntrophic benefit to their hosts under anoxic conditions, when the ciliates can yield energy only by fermentation. By assimilating fermentation waste products and keeping their concentrations low in their host, the symbionts can improve the energy yields for their hosts and allow them to better tolerate periods of anoxia. This could also be a form of resource recycling under carbon-limited conditions, which has been proposed for other thiotrophic symbionts with the potential to assimilate organic acids (3, 7).

Kentron is relatively enriched in ^13^C compared to nonsymbiotic shallow-water benthic fauna, such as nematodes and bivalves (δ-^13^C ∼ −20 to −10‰) (5, 60, 61), and to the seagrasses (δ-^13^C ∼ −15 to −10‰) (60–63) that are the main primary producers in the habitat of *Kentrophoros* (Fig. 6). The higher values in Kentron could be partly caused by preferring specific substrates with higher ^13^C content, such as acetate, which has a wide range of δ-^13^C (−2.8 to −20.7‰) in marine pore waters depending on the dominant microbial processes at the site (64). Given how close the δ-^13^C of Kentron is to DIC, it is possible that heterotrophic CO_2_ fixation contributes to this ^13^C signature, but the isotope fractionation values of the heterotrophic carboxylases have not been characterized, to our knowledge. Repeated internal recycling of host waste products, as we postulate, could also cause accumulation of ^13^C in the host-symbiont system.

Our metabolic model has parallels to free-living thiotrophs and to heterotrophic bacteria involved in enhanced biological phosphorus removal (EBPR) from wastewater, which also use storage inclusions like polyphosphate, glycogen, and PHA as metabolic buffers for fluctuating oxygen and nutrient conditions (65–68).

### Conclusion.

We have shown that a diverse and widespread clade of symbiotic sulfur bacteria lacks genes encoding canonical enzymes for autotrophic CO_2_ fixation, despite being a food source for their hosts. This is unlike all other thiotrophic symbionts sequenced to date, which possess the CBB or rTCA cycles for autotrophy. We propose a lithoheterotrophic model for the *Kentrophoros* nutritional symbiosis, which challenges the chemoautotrophic paradigm usually applied to thiotrophic symbiosis. Uptake of organic substrates from the environment, heterotrophic carboxylation, and recycling of host waste may play a bigger part in thiotrophic symbioses than previously thought. Our results suggest that nutritional symbioses can also be supported by chemolithoheterotrophy and that thiotrophic symbioses fall on a spectrum between autotrophy and heterotrophy. We speculate that heterotrophy is feasible for Kentron because its hosts are relatively small and flat and have the highest known symbiont-host biomass ratio among thiotrophs; larger hosts will experience more diffusion limitation and can fulfill only a smaller fraction of their carbon needs heterotrophically.

## MATERIALS AND METHODS

### Sample collection.

Specimens of *Kentrophoros* were collected in 2013 and 2014 from Elba, Italy (Mediterranean Sea); in 2015 from Twin Cayes, Belize (Caribbean Sea); and in 2016 from Nivå Bay, Denmark (Øresund Strait between Baltic and North Seas) (see data at https://doi.org/10.5281/zenodo.2575755), as previously described (20).

### Microscopy.

Live *Kentrophoros* sp. H was macerated to release bacteria and fixed with 4% (wt/vol) formaldehyde in seawater on glass slides. Cells were rinsed and stained with DAPI (1 μg/ml) and Nile Red (2.5 μg/ml) for 20 min, rinsed, and imaged under epifluorescence with a long-pass DAPI and fluorescein isothiocyanate (FITC)/enhanced green fluorescent protein (EGFP) long-pass filter cube, respectively, and a Canon EOS 700D camera.

### DNA/RNA extraction and sequencing.

Samples for DNA and RNA extraction, comprising single ciliate cells and their symbionts, were fixed in RNAlater (Ambion) and stored at 4°C. Before DNA extraction, samples were centrifuged (8,000 × *g*, 5 min), and excess RNAlater was removed by pipetting. DNA was extracted with the DNeasy Blood and Tissue kit (Qiagen) according to the manufacturer's instructions and eluted in 50 μl of buffer AE. DNA concentration was measured fluorometrically with the Qubit DNA high-sensitivity kit (Life Technologies). Each DNA sample was screened by PCR with eukaryotic 18S rRNA primers EukA/EukB (69) followed by capillary sequencing to identify the *Kentrophoros* phylotype, as previously described (20). Libraries for metagenomic sequencing were prepared with the Ovation Ultralow Library System V2 kit (NuGEN) according to the manufacturer's protocol. Libraries were sequenced as either 100- or 150-bp paired-end reads on the Illumina HiSeq 2500 platform.

RNA was extracted with the RNeasy Plus Micro kit (Qiagen) according to the manufacturer's protocol and eluted in 15 μl RNase-free water. cDNA was synthesized with the Ovation RNASeq System v2 (NuGEN) according to the manufacturer's protocol, sheared to 350-bp target size with the Covaris microTUBE system, and cleaned up with the Zymo Genomic DNA Clean & Concentrator kit. The sequencing library was prepared from cDNA with the NEBNext Ultra DNA library preparation kit for Illumina and sequenced on the Illumina HiSeq 2500 platform as 100-bp single-end reads.

### Assembly, binning, and annotation of symbiont genomes.

Reads were trimmed from both ends to remove fragments matching TruSeq adapters and to remove bases with Phred quality scores of <2, using either Nesoni v0.111 (https://github.com/Victorian-Bioinformatics-Consortium/nesoni) or BBmap v34+ (https://sourceforge.net/projects/bbmap/). Trimmed reads were error corrected with BayesHammer (70). Error-corrected reads were initially assembled with IDBA-UD v1.1.1 (71) or SPAdes v3.5.0+ (70). The reference coverage per contig was obtained by mapping the error-corrected read set against the assembly with BBmap (“fast” mode). Conserved marker genes in the assembly were identified and taxonomically classified with Amphora2 (72) or Phyla-Amphora (73). 16S rRNA genes were identified with Barrnap v0.5 (https://github.com/tseemann/barrnap) and classified by searching against the Silva SSU-Ref NR 119 database (74) with Usearch v8.1.1831 (75). Differential coverage information (76) was obtained by mapping reads from other samples of the same host morphospecies onto the assembly with BBmap. Contigs belonging to the primary *Kentrophoros* symbiont (the “primary symbiont bin”) were heuristically identified by a combination of differential coverage, assembly graph connectivity, GC%, affiliation of conserved marker genes, and affiliation of 16S rRNA sequence using gbtools v2.5.2 (77). Reads mapping to the primary symbiont bin were reassembled with SPAdes. Binning and reassembly of the primary symbiont genome were iteratively repeated for each metagenome sample until the primary symbiont bin appeared to contain only a single genome, based on the number and taxonomic affiliation of conserved marker genes and 16S rRNA. For final genome bins, summary statistics were computed with Quast v4.4 (78), and completeness and contamination were estimated with CheckM v1.0.11 (79) using the *Gammaproteobacteria* taxonomy workflow. Average amino acid identity (AAI) and average nucleotide identity (ANI) values between genomes were calculated with CompareM v0.0.21 (https://github.com/dparks1134/CompareM) and jSpecies v1.2.1, respectively (80).

Genome bins were annotated with the IMG/M pipeline for downstream analyses (81). Metabolic pathways were predicted from the annotated proteins with the PathoLogic module (82) of Pathway Tools v20.5 (83), followed by manual curation. Metabolic modules from KEGG (84) were also predicted with the KEGG Mapper tool (http://www.kegg.jp/kegg/mapper.html, accessed January 2017) from KEGG Orthology terms in the IMG annotation.

### Transcriptome analysis.

Metatranscriptome reads for *Kentrophoros* sp. H and SD were mapped onto symbiont genome assemblies from the respective species (IMG genome IDs 2609459750 and 2615840505) using BBmap (minimum identity, 0.97). Read counts per genomic feature were calculated with featureCounts v1.5.2 (85) and transformed into FPKM values (fragments per kilobase pair reference per million reads mapped).

### Verifying absence of key genes for autotrophic pathways.

Key enzymes that are diagnostic for known autotrophic pathways were identified from the literature (48, 86–88) (see data posted at https://doi.org/10.5281/zenodo.2575773). These were absent from Kentron genome annotations, with the exception of a RuBisCO-like protein (RLP) in Kentron sp. H (see below). To verify that the absence of autotrophy-related sequences was not caused by incomplete genome bins, misprediction of open reading frames, or misassembly of the reads, we aligned raw reads from host-symbiont metagenomes and metatranscriptomes against the UniProt Swiss-Prot database (release 2017_01) (89) using diamond blastx (v0.8.34.96, “sensitive” mode) (90). Sequences for certain key enzymes were absent from Swiss-Prot, so representative sequences from UniProtKB were manually added to the database (see data posted at https://doi.org/10.5281/zenodo.2575773 and File 4 at https://doi.org/10.5281/zenodo.2555833). Reads with hits to target enzymes (identified by EC number or from the list of additional sequences) were counted, extracted, and mapped against the initial metagenomic assembly for the corresponding library. Raw counts of reads were transformed to FPKM values using three times the mean amino acid length of the target proteins as the reference length. As a comparison, FPKM values were also calculated for a reference set of enzymes of the TCA cycle and partial 3HPB pathway (see data posted at https://doi.org/10.5281/zenodo.2575773), which were expected to be present in all Kentron genomes.

### Identification of transporter genes for substrate uptake.

Families and subfamilies of transporter proteins from the Transporter Classification Database (TCDB, accessed 2 February 2017) (91) that were described as energy-dependent uptake transporters for organic substrates were shortlisted (see data posted at https://doi.org/10.5281/zenodo.2575779). Translated open reading frames (ORFs) for Kentron and selected genomes of other symbiotic and free-living basal *Gammaproteobacteria* (see data posted at https://doi.org/10.5281/zenodo.2575781) were aligned with BLASTP (92) (best-scoring hit with E value of <10^−5^, >30% amino acid sequence identity, and >70% coverage of reference sequence, parameters from reference 55) against TCDB. As TCDB also includes nonmembrane proteins that are involved in transport (e.g., the ATPase subunit of ABC transporters), we also counted how many hits contained transmembrane domains, predicted with tmhmm v2.0c (93). To compare the transporter content between genomes, the tabulated counts of organic substrate uptake TC family hits per genome were analyzed by nonmetric multidimensional scaling (NMDS) with the metaMDS function in the R package vegan v2.5.1 (https://CRAN.R-project.org/package=vegan) (Bray-Curtis distance, 2 dimensions, 2,000 runs).

### Protein extraction and peptide preparation.

Samples of *Kentrophoros* sp. H for proteomics were collected by decantation from sediment adjacent to seagrass meadows at Sant’ Andrea, Isola d’Elba, Italy on 3 June 2014 and from Pampelonne Beach, Provence-Alpes-Côte d’Azur, France, in July 2018. Ciliates were individually fixed in RNAlater and subsequently stored at 4°C and then at −80°C. One individual *Kentrophoros* sp. H specimen and nine pooled samples of four or five individuals each (see data posted at https://doi.org/10.5281/zenodo.2575800) were used to prepare tryptic digests following the filter-aided sample preparation (FASP) protocol (94) with minor modifications (95). Samples were lysed in 30 μl of SDT-lysis buffer (4% [wt/vol] SDS, 100 mM Tris-HCl pH 7.6, 0.1 M DTT) by heating to 95°C for 10 min. To avoid sample losses, we did not clear the lysate by centrifugation after lysis. Instead, we loaded the whole lysate on to the 10-kDa filter units used for the FASP procedure. The Qubit protein assay kit (Thermo Fisher Scientific, Life Technologies) was used to determine peptide concentrations, according to the manufacturer’s instructions. Peptide concentrations were below the detection limit in all samples.

### 1D-LC-MS/MS.

All peptide samples were analyzed by 1D-LC-MS/MS as previously described (96), with the modification that a 75-cm analytical column was used. Briefly, an UltiMate 3000 RSLCnano liquid chromatograph (Thermo Fisher Scientific) was used to load peptides with loading solvent A (2% acetonitrile, 0.05% trifluoroacetic acid) onto a 5-mm, 300-μm-internal-diameter (i.d.) C_18_ Acclaim PepMap100 precolumn (Thermo Fisher Scientific). Since peptide concentrations were very low, complete peptide samples (80 μl) were loaded onto the precolumn. Peptides were eluted from the precolumn onto a 75-cm by 75-μm analytical EASY-Spray column packed with PepMap RSLC C_18_, 2-μm material (Thermo Fisher Scientific) heated to 60°C. Separation of peptides on the analytical column was achieved at a flow rate of 225 nl min^−1^ using a 460-min gradient going from 98% buffer A (0.1% formic acid) to 31% buffer B (0.08% formic acid, 80% acetonitrile) in 363 min and then to 50% B in 70 min and to 99% B in 1 min and ending with 26 min 99% B. Eluting peptides were analyzed in a Q Exactive Plus hybrid quadrupole-Orbitrap mass spectrometer (Thermo Fisher Scientific). Carryover was reduced by running two wash runs (injection of 20 μl acetonitrile) between samples. Data acquisition in the Q Exactive Plus was done as previously described (5).

### Protein identification and quantification.

Protein sequences predicted from Kentron genomes described above were clustered at 98% identity with CD-HIT v4.7 (97). Representative sequences, proteins predicted from a preliminary host transcriptome, and the cRAP protein sequence database (http://www.thegpm.org/crap/) of common lab contaminants were used as a database for protein identification, which contained 5,715 protein sequences. For protein identification, MS/MS spectra were searched against this database using the Sequest HT node in Proteome Discoverer version 2.2 (Thermo Fisher Scientific) as previously described (5).

### Direct protein-SIF.

Stable carbon isotope fingerprints (SIFs = δ-^13^C values) for Kentron symbiosis were determined from proteomic data (37). Briefly, human hair with a known δ-^13^C value was used as reference material to correct for instrument fractionation. A tryptic digest of the reference material was prepared as described above and analyzed with the same 1D-LC-MS/MS method as the samples. The peptide-spectrum match (PSM) files generated by Proteome Discoverer were exported in tab-delimited text format. 1D-LC-MS/MS raw files were converted to mzML format using the MSConvertGUI available in the ProteoWizard tool suite (98). Only the MS^1^ spectra were retained in the mzML files, and the spectra were converted to centroided data by vendor algorithm peak picking. The PSM and mzML files were used as input for the Calis-p software (https://sourceforge.net/projects/calis-p/) to extract peptide isotope distributions and to compute the direct protein-SIF δ-^13^C value for Kentron and the human hair reference material (37). Direct protein-SIF δ-^13^C values were corrected for instrument fragmentation by applying the offset between the direct protein-SIF δ-^13^C value and known δ-^13^C value of the reference material. We obtained between 50 and 499 peptides with sufficient intensity for direct protein-SIF from seven of the nine pooled samples (see data posted at https://doi.org/10.5281/zenodo.2575800). These samples were thus well above the necessary number of peptides needed to obtain an accurate estimate. Due to the low biomass of the individual *Kentrophoros* specimen (∼10 μg), only 14 peptides with sufficient intensity were obtained for this sample.

### Dissolved inorganic carbon δ-^13^C.

Seawater and pore water samples were collected from the vicinity of seagrass meadows at Sant’ Andrea, Elba, Italy, in July 2017 to determine the δ-^13^C of dissolved inorganic carbon (DIC). Seawater was sampled at the surface from a boat, whereas pore water was sampled at 15-cm sediment depth with a steel lance. Samples were drawn into 20-ml plastic syringes; 6 ml of each was fixed with 100 μl of 300 mM ZnCl_2_ and stored at 4°C until processing. δ-^13^C was measured with a Finnigan MAT 252 gas isotope ratio mass spectrometer with Gasbench II (Thermo Scientific), using Solnhofen limestone as a standard and 8 technical replicates per sample.

### Code availability.

Scripts used to screen for autotrophy-related genes in metagenome libraries, to classify transporter families, and to calculate phylogenetic trees are available at https://github.com/kbseah/mapfunc, https://github.com/kbseah/tcdbparse_sqlite, and https://github.com/kbseah/phylogenomics-tools, respectively.

### Data availability.

Annotated genomes are in the Joint Genome Institute GOLD database (https://gold.jgi.doe.gov/) study Gs0114545. Metagenomes and metatranscriptomes are in European Nucleotide Archive studies PRJEB25374 and PRJEB25540. The mass spectrometry metaproteomics are in ProteomeXchange Consortium via the PRIDE partner repository (https://www.ebi.ac.uk/pride/archive/) data set PXD011616.
