# Supplementary material for: Sulfur-Oxidizing Symbionts without Canonical Genes for Autotrophic CO2 Fixation
Source: mBio. 2019 Jun 25;10(3):e01112-19. doi: 10.1128/mBio.01112-19 (PMC6593406; doi:10.1128/mBio.01112-19)
Supplement: TEXT S1 [file mBio.01112-19-s0001.pdf]

Supplementary Text 1
Phylogenetics

to accompany

Sulfur-oxidizing symbionts without canonical genes for autotrophic CO2 fixation

Brandon K. B. Seah, Chakkiath Paul Antony, Bruno Huettel, Jan Zarzycki, Lennart Schada von Borzyskowski, Tobias J. Erb, Angela Kouris, Manuel Kleiner, Manuel Liebeke, Nicole Dubilier, Harald R. Gruber-Vodicka

Supplementary Results and Discussion

Phylogenetic position and diversity of the Kentron clade

Kentron formed a well-supported clade (100% SH-like support value) within the Gammaproteobacteria, in a phylogenetic analysis using conserved protein-coding marker genes. Their closest relatives in the set of basal Gammaproteobacteria analysed were *Nitrosococcus oceani*, *Methylophaga thiooxydans*, *Thioploca ingrica*, *Ca*. Competibacter denitrificans, and *Beggiatoa* spp. (100% support), which differed from the 16S rRNA gene phylogeny, where Kentron was sister to the Coxiellaceae (1). Symbionts from different host morphospecies formed separate, well-supported phylotype clusters, with the exception of Kentron from *Kentrophoros* sp. UNK and *K*. sp. LPFa, where a single symbiont phylotype was associated with two different host phylotypes, as previously observed with 16S and 18S rRNA sequences. Among genomes of the same phylotype, average nucleotide identities *(*ANI*)* were 93.0–100% and average amino acid identities (AAI) were 93.2–100%, whereas between different phylotypes, these values were 83.2–93.8% and 70.6–91.3% respectively, which supports them being different species in the same genus (2). Kentron phylotypes will therefore be referred to here with their corresponding host morphospecies identifiers, except for Kentron UNK/LPFa.

Supplementary Materials and Methods

Core- and pan-genome analysis

Ortholog clusters of Kentron protein sequences were predicted by first performing a reciprocal Blastp (version 2.2.29+) search (3) of all translated open reading frames (ORFs) annotated by the IMG pipeline (E-value cutoff 10^{-5}), and then identifying clusters in the search results with the Markov cluster algorithm (4) using FastOrtho (inflation value 1.5), which is a reimplementation of OrthoMCL (5) by the PATRIC project (6). Accumulation curves and uncertainty estimates for the core and pan genome size were generated by random resampling (n = 200) of genome memberships for the predicted orthologs.

Phylogenetic analyses

Maximum-likelihood phylogenetic trees were inferred from the following alignments with Fasttree v2.1.7 (7), using the JTT model with CAT approximation (20 rate categories) and SH-like support values.

Kentron and related Gammaproteobacteria. Conserved marker genes from Kentron and selected basal Gammaproteobacteria (https://doi.org/10.5281/zenodo.2575781) were extracted by the Amphora2 pipeline. Amino acid sequences of 30 markers were aligned with Muscle v3.8.31 (8) and concatenated.

RuBisCO-like protein from Kentron sp. H. RuBisCO superfamily protein accessions and their classification were obtained from (9). These were aligned with RuBisCO-like protein sequences from Kentron sp. H and RuBisCO from selected sulfur-oxidizing symbiotic Gammaproteobacteria, using Muscle.

Proteins of partial 3-hydroxypropionate bi-cycle. Homologs to proteins of the 3-hydroxypropionate bi-cycle in *Chloroflexus aurantiacus* were obtained from the UniRef50 clusters containing the *C. aurantiacus* sequences in the UniProt database. These were aligned with the Kentron homologs with Muscle.

References

1. Seah BKB, Schwaha T, Volland J-M, Huettel B, Dubilier N, Gruber-Vodicka HR. 2017. Specificity in diversity: single origin of a widespread ciliate-bacteria symbiosis. Proceedings of the Royal Society B: Biological Sciences 284:20170764.

2. Rodriguez-R LM, Konstantinidis KT. 2014. Bypassing cultivation to identify bacterial species. Microbe 9:111–8.

3. Camacho C, Coulouris G, Avagyan V, Ma N, Papadopoulos J, Bealer K, Madden TL. 2009. BLAST+: architecture and applications. BMC Bioinformatics 10:421.

4. van Dongen S, Abreu-Goodger C. 2012. Using MCL to extract clusters from networks, p. 281–295. *In* van Helden, J, Toussaint, A, Thieffry, D (eds.), Bacterial Molecular Networks. Springer New York, New York, NY.

5. Li L, Stoeckert CJ, Roos DS. 2003. OrthoMCL: identification of ortholog groups for eukaryotic genomes. Genome research 13:2178–2189.

6. Wattam AR, Abraham D, Dalay O, Disz TL, Driscoll T, Gabbard JL, Gillespie JJ, Gough R, Hix D, Kenyon R, Machi D, Mao C, Nordberg EK, Olson R, Overbeek R, Pusch GD, Shukla M, Schulman J, Stevens RL, Sullivan DE, Vonstein V, Warren A, Will R, Wilson MJC, Yoo HS, Zhang C, Zhang Y, Sobral BW. 2014. PATRIC, the bacterial bioinformatics database and analysis resource. Nucleic Acids Research 42:D581–D591.

7. Price MN, Dehal PS, Arkin AP. 2010. FastTree 2–approximately maximum-likelihood trees for large alignments. PloS one 5:e9490.

8. Edgar RC. 2004. MUSCLE: multiple sequence alignment with high accuracy and high throughput. Nucleic Acids Research 32:1792–1797.

9. Tabita FR, Hanson TE, Li H, Satagopan S, Singh J, Chan S. 2007. Function, structure, and evolution of the RubisCO-Like Proteins and their RubisCO homologs. Microbiology and Molecular Biology Reviews 71:576–599.
