# Supplementary figures and images for: Sulfur-Oxidizing Symbionts without Canonical Genes for Autotrophic CO2 Fixation
Source: mBio. 2019 Jun 25;10(3):e01112-19. doi: 10.1128/mBio.01112-19 (PMC6593406; doi:10.1128/mBio.01112-19)

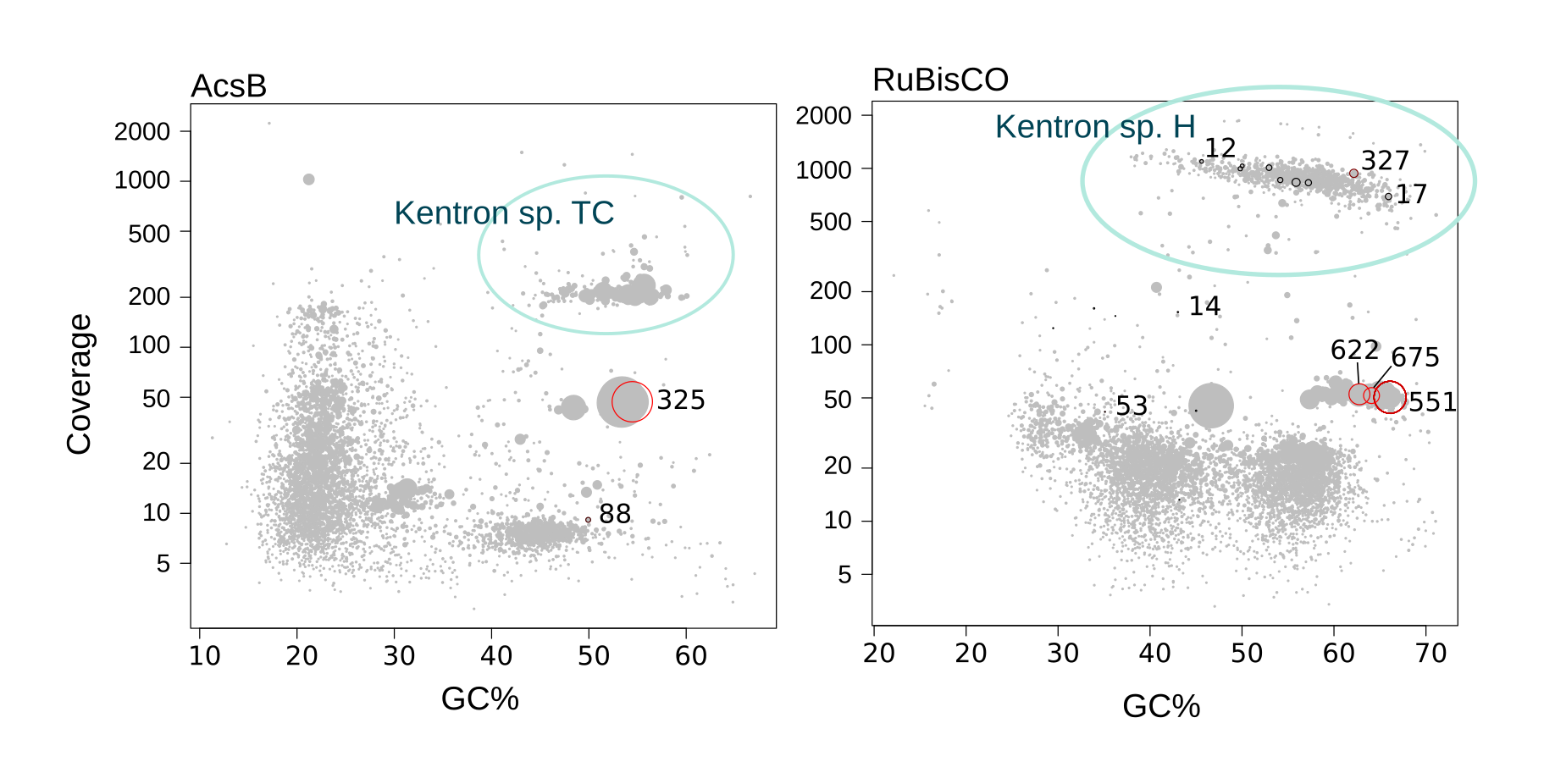

Supplement: FIG S3 [file mBio.01112-19-sf003.tif]

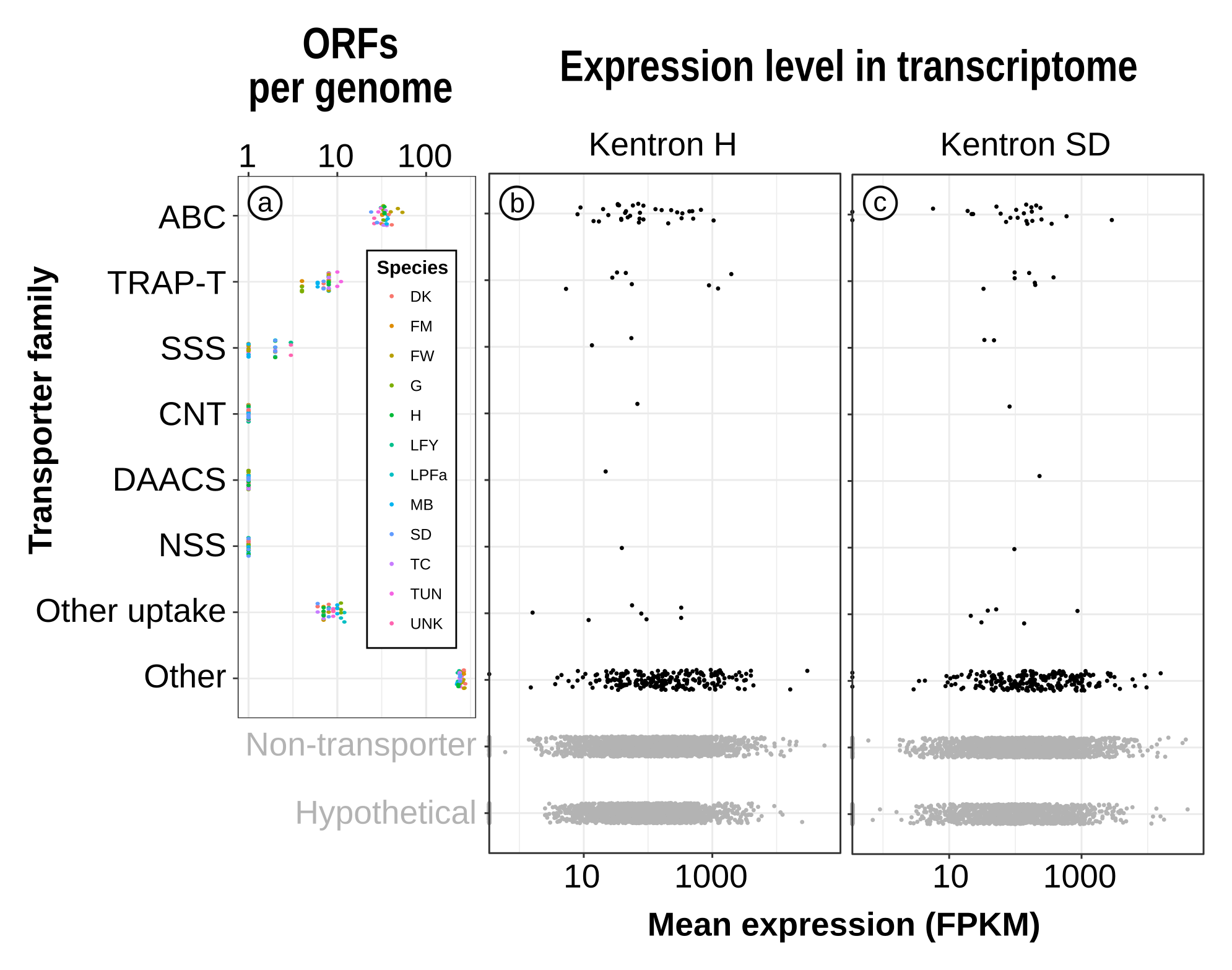

Supplement: FIG S5 [file mBio.01112-19-sf005.tif]
