# Supplementary material for: Sulfur-Oxidizing Symbionts without Canonical Genes for Autotrophic CO2 Fixation
Source: mBio. 2019 Jun 25;10(3):e01112-19. doi: 10.1128/mBio.01112-19 (PMC6593406; doi:10.1128/mBio.01112-19)
Supplement: TEXT S2 [file mBio.01112-19-s0002.pdf]

Supplementary Text 2
Metabolomics

to accompany

Sulfur-oxidizing symbionts without canonical genes for autotrophic CO2 fixation

Brandon K. B. Seah, Chakkiath Paul Antony, Bruno Huettel, Jan Zarzycki, Lennart Schada von Borzyskowski, Tobias J. Erb, Angela Kouris, Manuel Kleiner, Manuel Liebeke, Nicole Dubilier, Harald R. Gruber-Vodicka

Supplementary Results and Discussion

Metabolites detected in Kentrophoros

Kentrophoros sp. H was used for metabolite profiling as it was the largest morphospecies known to us, and could be reliably collected from one site. The predominant metabolite detected was the disaccharide trehalose. Sucrose was also detected, but in smaller quantities. Other possible metabolites detected were also found in the blanks and hence disregarded. Trehalose is a disaccharide of glucose that is phylogenetically widespread, found in both eukaryotes and prokaryotes (1). Only two Kentron phylotypes have the potential for trehalose synthesis, via trehalose synthase, but none have pathways for trehalose breakdown (e.g. trehalase) nor PTS-type sugar uptake transporters. Therefore, trehalose is likely produced and stored by the host ciliates, where they could function as either an energy store or an osmoprotectant.

Supplementary Materials and Methods

Metabolite extraction and identification

Kentrophoros sp. H was collected on Elba in 2014 for metabolomics (https://doi.org/10.5281/zenodo.2575783). Samples were fixed in 1 mL cold methanol (HPLC-grade, Sigma-Aldrich) and stored at -20°C until use. Ribitol (40 µL, 200 mg L^{-1}, aqueous) was added as internal standard. For metabolite extraction, each sample was resuspended by vortexing,

transferred to a bead-beating vial (FastPrep Lysing Matrix B, MP Biomedicals), and disrupted (4 ms^{-1}, 40 s). The vial was centrifuged (16000 rcf, 2 min) and supernatant transferred to a new tube. AMW mixture (1 mL acetonitrile/methanol/water in 2:2:1 ratio) was added to the bead-beating vial, which was disrupted and centrifuged again. Supernatant containing metabolites was pooled, and evaporated to dryness under vacuum (Concentrator Plus, Eppendorf, V-AL mode, 30°C, 4 h).

Dried samples were derivatized with methoxyamine hydrochloride (MeOX) dissolved in pyridine and N,O-bis(trimethylsilyl)trifluoroacetamide with 1% trimethylchlorosilane (BSTFA + 1% TMCS). For each sample, MeOX (80 µL, 20 mg mL^{-1}) was added, briefly vortexed, and then heated with shaking (37 °C, 1200 rpm, 90 min). The pyridine was evaporated under a stream of N_2 gas until samples were dry (≥1 h). 80 µL of BSTFA (Chromatographie Service) was added, vortexed, and heated with shaking (37 °C, 1350 rpm, 30 min). Samples were briefly centrifuged down and transferred to glass vials for GC-MS (Insert G27, spring S27, Mikro-KH-Vial G1; Chromatographie Service). GC-MS analysis was performed on a 7890B GC system (Agilent Technologies) coupled to a 5977A MSD (Agilent). He was used as carrier gas at constant flow of 1 mL min^{-1}. The temperature program was 60 °C (2min), increase to 300 °C at 10 °C min^{-1}, hold at 325 °C (7 min). The quadrupole MS was operated in electron ionization mode at 70 eV, with scanning range set to 50-600 m/z.

GC-MS data were screened with AMDIS software for known metabolites. Mass spectra were deconvoluted with the AMDIS algorithm ("simple" mode), and searched against an in-house database. Quantification was performed with Agilent Quantiative Analysis software. A quantitation method was created from acquired scan data with the built-in deconvolution algorithm, using default settings except that m/z value 73 was excluded as a quantifier because it corresponds to a derivitization product (trimethylsilyl group). Predicted compounds were assigned identities based on matches to the NIST database and/or in-house database. The method was applied to all samples and blanks as a batch, with default values except GC retention time window of 0.10 min. Quantifier

and qualifier peaks were manually curated to correct misassigned peaks. Compounds present in both blanks and samples in similar quantities were excluded.

References

1. Elbein AD, Pan YT, Pastuszak I, Carroll D. 2003. New insights on trehalose: a multifunctional molecule. Glycobiology 13:17R-27R.
