# Supplementary material for: Sulfur-Oxidizing Symbionts without Canonical Genes for Autotrophic CO2 Fixation
Source: mBio. 2019 Jun 25;10(3):e01112-19. doi: 10.1128/mBio.01112-19 (PMC6593406; doi:10.1128/mBio.01112-19)
Supplement: TEXT S3 [file mBio.01112-19-s0003.pdf]

Supplementary Text 3
Metabolic reconstruction details

to accompany

Sulfur-oxidizing symbionts without canonical genes for autotrophic CO2 fixation

Brandon K. B. Seah, Chakkiath Paul Antony, Bruno Huettel, Jan Zarzycki, Lennart Schada von Borzyskowski, Tobias J. Erb, Angela Kouris, Manuel Kleiner, Manuel Liebeke, Nicole Dubilier, Harald R. Gruber-Vodicka

Supplementary Results and Discussion

Reactions that potentially allow for autotrophic CO_2 fixation

Although no known pathways for autotrophic CO_2 fixation were predicted in Kentron genomes, several enzymes predicted in Kentron genomes can potentially catalyze a network of reactions that would allow autotrophic CO_2 fixation, by combining reactions of the 3-hydroxypropionate bi-cycle (3HPB) (1) and a hypothetical pathway that was previously proposed for *Chloroflexus* (2). The original version of the proposed Ivanovsky pathway by itself would have presented a metabolic dead end, as its net product is glyoxylate, and the only other predicted enzyme in Kentron that metabolizes glyoxylate, malate synthase, effectively reverses the last step of the Ivanovsky pathway. Other reactions that could convert glyoxylate to downstream metabolites, such as tartronate semialdehyde synthase, or isocitrate lyase (part of the glyoxylate shunt), were not predicted for Kentron. However, by allowing the interconversion between the reactant pairs acetyl-CoA/pyruvate and propionyl-CoA/glyoxylate via C5 intermediates in reactions that were previously thought to be unique to the 3HPB, it is possible to form a closed cycle whose net product is pyruvate (https://doi.org/10.5281/zenodo.2575789).

Some of these reactions would be catalyzed by alternative enzymes compared to the versions originally presented in (2) or (3): PEP carboxykinase (GDP) instead of PEP carboxylase, succinyl-CoA:malate CoA transferase instead of malyl-CoA synthetase, and pyruvate phosphate dikinase

instead of pyruvate water kinase. Genes for all the components of the hypothetical pathway were expressed in the transcriptomes sequenced (https://doi.org/10.5281/zenodo.2555833, File 1). The putative methylmalonyl-CoA epimerases were originally annotated as lactoylglutathione lyase in IMG, but as the sequences are relatively short (ca. 150 a.a.), they were re-evaluated as likely methylmalonyl-CoA epimerases because their genes were frequently adjacent to those for acyl-CoA carboxyltransferases, and an InterPro search found signatures such as the VOC domain (InterPro IPR037523) and Glyoxalase_4 domain (Pfam PF13669) which are also characteristic of methylmalonyl-CoA epimerase, but not the signatures characteristic of lactoylglutathione lyase (e.g. IPR019883 and IPR004361).

Thermodynamic favorability of the proposed reactions

The overall net reaction is exergonic (-60.9 kJ/mol), but some individual steps are endergonic and could be significant barriers. The most positive $\Delta_r G'^m$ values are for pyruvate synthase (18.7 kJ/mol) and pyruvate phosphate dikinase (19.6 kJ/mol), whereas carboxylation of PEP requires 4.3 kJ/mol. However, for the phosphorylation of pyruvate, the by-product pyrophosphate is very favorably hydrolyzed to two orthophosphate groups, and the net reaction is exergonic (-13.3 kJ/mol) and would be favored if they were coupled. The hydrolysis of pyrophosphate can be coupled to energy conservation by Na^+/H^+-translocating pyrophosphatase, which is predicted in all Kentron genomes.

The pyruvate synthase reaction can also be more favorable if the ratio of reduced:oxidized ferredoxin (assuming reduction potential of -418 +/- 60 mV) in the cell is on the order of 100-fold or greater (https://doi.org/10.5281/zenodo.2575791). The value of +18.7 kJ/mol is for 1 mM concentrations of both reduced and oxidized species. At a ratio of 100:1, the reaction could be exergonic (although the uncertainty is +/-13.2 kJ/mol). Such an “over-reduced” state in the cell could be maintained by the Rnf-type Na^+-translocating oxidoreductases, which can reduce ferredoxin with NADH using energy from a Na^+ membrane gradient. These are predicted in Kentron genomes, and are relatively common among facultatively anaerobic bacteria (4).

In kinetic terms, however, pyruvate synthase is slow (specific activity < 0.1 to 2.3 µmol min^{-1} mg^{-1}) and has low substrate specificity (K_M 2 to 48 mM) in organisms where these parameters have been measured; in comparison, RuBisCO has a specific activity of 2 to 4 µmol min^{-1} mg^{-1} (cited in (3)). This means that pyruvate synthase would have to be highly expressed, at a level comparable to RuBisCO in CBB-cycle organisms, for it to be an effective autotrophic carboxylase. In Kentron H, pyruvate synthase was among the top 5% in expression level (Figure 4), but this was only about 6% of the expression level of the top-expressed gene (a predicted phasin). In comparison, RuBisCO is typically the most-expressed, or among the most-expressed genes in thiotroph transcriptomes (5, 6). Nonetheless, as the organisms had to be extracted from their natural sediment habitat before being fixed, it is likely that the gene expression levels do not reflect their *in situ* metabolic states.

Role of these reactions in Kentron and other bacteria

Other bacterial genomes that encode enzymes for an incomplete 3HPB, namely *Ca*. Thiosymbion and *Ca*. Accumulibacter, also encoded genes for other enzymes of the hypothetical pathway (https://doi.org/10.5281/zenodo.2555833, File 1). *Ca*. Thiosymbion are is a thiotrophic symbionts like Kentron, but they already possess a functioning CBB cycle (7). In comparison, *Ca*. Accumulibacter are chemoorganotrophs from wastewater treatment plants, and it is unclear why they would possess an autotrophic pathway (some *Ca*. Accumulibacter also encode a CBB cycle).

Like the complete 3HPB in *Chloroflexus* (8), the hypothetical pathway would also allow the co-assimilation of organic substrates, such as succinate, malate, and propionate. Given that (i) such organic acids are predicted substrates of the uptake transporters encoded in Kentron genomes and are also common in coastal sediments, (ii) the reactions are also predicted in a thiotrophic symbiont that uses the CBB cycle for autotrophy, and (iii) the 3HPB is used mixotrophically in *Chloroflexus*, it is likely that the usual nutritional mode of Kentron is lithoheterotrophic, or at most mixotrophic. If CO_2 fixation only occurs with organic co-assimilation, it would then be indistinguishable from heterotrophic CO_2 fixation, especially as the carboxylases involved are also typical for carboxylation in heterotrophs.

Oxidation/reduction value of substrates and biomass

The oxidation/reduction (O/R) value can be used as a measure of how oxidized or reduced a substrate is relative to biomass, and hence whether additional reducing equivalents are required for its assimilation (9). For a molecular formula where the ratio of H:O is x:y, the oxidation/reduction level $r = (2y - x)/2$. The empirical formula $CH_{1.77}O_{0.49}N_{0.24}$ of biomass for *Escherichia coli* was used (10), which is close to empirical formulas for other bacteria, e.g. the purple non-sulfur bacterium *Rhodopseudomonas palustris* $CH_{1.8}O_{0.38}N_{0.18}$ (11). O/R values for potential substrates and storage compounds of Kentron are shown here: https://doi.org/10.5281/zenodo.2575777. Assimilation of malate, succinate, acetate and the mobilization of glycogen for biosynthesis would require additional reducing equivalents, whereas polyhydroxybutyrate (a form of PHA) could serve as a store of reducing equivalents.

Occurrence of lithoheterotrophic metabolism in thiotroph genomes

A total of 1407 thiotrophic bacterial genomes in the IMG/ER database (excluding Kentron) were predicted to encode lithoheterotrophic metabolism, based on a screening using key genes for sulfur oxidation and autotrophy (CBB and rTCA cycles). Genomes that encode the rDsr/Sox pathway but lack either CBB or rTCA cycles are uncommon – only seven genomes were found (https://doi.org/10.5281/zenodo.2575796). One of these, *Magnetococcus marinus* MC-1 was a false negative, as it has been reported to use the rTCA cycle (12), but it encodes a variant of the ATP citrate lyase that was not annotated with a KO number by the IMG pipeline. The remainder included two isolates – *Ruegeria marina* CGMCC 1.9108, *Thiothrix flexilis* DSM 14609 – and four genome bins from environmental metagenomes: *Thioalkalivibrio* spp. HK1 and TsSOB (both associated with sponges), REDSEA-S14_B17, and REDSEA-S15_B12. Most genomes that encode the rDsr/Sox pathway have either the CBB cycle (89 genomes) or both (8). In contrast, those that encode the Sox pathway only are roughly as likely to have at least one autotrophic pathway (642) as not (661).

The Sox pathway alone allows oxidation of reduced sulfur in the form of thiosulfate. Additional enzymes (Sqr and FccAB) would also allow oxidation of sulfide. However, bacteria that can store elemental sulfur or polysulfide as cellular inclusions typically have the rDsr/Sox pathway, because the Dsr components are required to mobilize the stored sulfur (13). We hypothesize that the rDsr/Sox pathway is more often associated with autotrophic pathways because CO_2 fixation can secondarily serve as an additional electron sink under reducing conditions, when other electron acceptors such as oxygen or nitrate are unavailable.

Supplementary Materials and Methods

Screening for hypothetical autotrophic pathway genes in bacterial genomes

Five (meta)genomes known to have an incomplete 3HPB pathway were screened for genes that could hypothetically allow for autotrophic CO_2 fixation, using the Gene Profile tool in IMG/ER (unidirectional sequence similarities, Blastp cutoffs at 10% identity, E-value < 0.1): *Ca*. Thiosymbion from *Olavius algarvensis*, *Ca*. Accumulibacter, and the Pink Berry consortium metagenome). Sequences from two Kentron genomes were used as queries. Nine other genomes belonging to other thiotrophic symbiotic bacteria were also screened with the same criteria. Accession numbers are given in https://doi.org/10.5281/zenodo.2555833, File 1.

Screening for lithoheterotrophic metabolism in bacterial genomes

Genomes available on the IMG/ER platform were screened for genes related to thiotrophic carbon metabolism and carbon fixation (CBB and rTCA cycles), using the following KEGG Orthology (KO) numbers. For sulfur oxidation: K11180, K11181, K17230, K17229, K17222, K17224, K17225, K17223, K17226, K17227, K17218. For CBB and rTCA cycles: K15230, K15231, K15234, K01601, K01602. For each KO term, the list of genomes containing a gene annotated with that KO number was retrieved, filtered to domain Bacteria, and including all genomes “All Finished, Permanent Draft, and Draft”.

Both reductive and oxidative (reverse) DsrAB are included under the same KO numbers. To distinguish between the two, the DsrAB amino acid sequences were downloaded from IMG, and aligned by Blastp against a database of DsrAB sequences that have been classified into oxidative and reductive types (14). The best hit was used to annotate the query sequences. However, a strain of *Desulfovibrio alkaliphilus* with reductive-type DsrAB (based on sequence homology) has recently been shown to be able to run the pathway in the oxidative direction, so the pathway may be more flexible than previously thought (15).

Incomplete genomes may give a false positive result of lithoheterotrophy. For the set of candidate lithoheterotrophs with the composite rDsr-Sox pathway, completeness was estimated with the CheckM pipeline (lineage workflow, reduced tree), and genomes with <75% estimated completeness were excluded.

References

1. Zarzycki J, Brecht V, Müller M, Fuchs G. 2009. Identifying the missing steps of the autotrophic 3-hydroxypropionate CO_2 fixation cycle in *Chloroflexus aurantiacus*. Proceedings of the National Academy of Sciences 106:21317–21322.

2. Ivanovsky RN, Krasilnikova EN, Fal YI. 1993. A pathway of the autotrophic CO_2 fixation in *Chloroflexus aurantiacus*. Arch Microbiol 159:257–264.

3. Bar-Even A, Noor E, Milo R. 2012. A survey of carbon fixation pathways through a quantitative lens. Journal of Experimental Botany 63:2325–2342.

4. Biegel E, Schmidt S, González JM, Müller V. 2011. Biochemistry, evolution and physiological function of the Rnf complex, a novel ion-motive electron transport complex in prokaryotes. Cell Mol Life Sci 68:613–634.

5. Seston SL, Beinart RA, Sarode N, Shockey AC, Ranjan P, Ganesh S, Girguis PR, Stewart FJ. 2016. Metatranscriptional response of chemoautotrophic *Ifremeria nautilei* endosymbionts to differing sulfur regimes. Front Microbiol 7:1074.

6. Stewart FJ, Dmytrenko O, DeLong EF, Cavanaugh CM. 2011. Metatranscriptomic analysis of sulfur oxidation genes in the endosymbiont of *Solemya velum*. Frontiers in Microbiology 2:134.

7. Kleiner M, Wentrup C, Lott C, Teeling H, Wetzel S, Young J, Chang Y-J, Shah M, VerBerkmoes NC, Zarzycki J, Fuchs G, Markert S, Hempel K, Voigt B, Becher D, Liebeke M, Lalk M, Albrecht D, Hecker M, Schweder T, Dubilier N. 2012. Metaproteomics of a gutless marine worm and its symbiotic microbial community reveal unusual pathways for carbon and energy use. Proceedings of the National Academy of Sciences 109:E1173–E1182.

8. Zarzycki J, Fuchs G. 2011. Coassimilation of organic substrates via the autotrophic 3-hydroxypropionate bi-cycle in *Chloroflexus aurantiacus*. Applied and Environmental Microbiology 77:6181–6188.

9. Gottschalk G. 1986. Bacterial Metabolism. Springer New York, New York, NY.

10. Grosz R, Stephanopoulos G. 1983. Statistical mechanical estimation of the free energy of formation of *E. coli* biomass for use with macroscopic bioreactor balances. Biotechnol Bioeng 25:2149–2163.

11. McKinlay JB, Harwood CS. 2010. Carbon dioxide fixation as a central redox cofactor recycling mechanism in bacteria. PNAS 107:11669–11675.

12. Williams TJ, Zhang CL, Scott JH, Bazylinski DA. 2006. Evidence for autotrophy via the reverse tricarboxylic acid cycle in the marine magnetotactic coccus strain MC-1. Applied and Environmental Microbiology 72:1322–1329.

13. Ghosh W, Dam B. 2009. Biochemistry and molecular biology of lithotrophic sulfur oxidation by taxonomically and ecologically diverse bacteria and archaea. FEMS Microbiol Rev 33:999–1043.

14. Müller AL, Kjeldsen KU, Rattei T, Pester M, Loy A. 2015. Phylogenetic and environmental diversity of DsrAB-type dissimilatory (bi)sulfite reductases. The ISME Journal 9:1152–1165.

15. Thorup C, Schramm A, Findlay AJ, Finster KW, Schreiber L. 2017. Disguised as a sulfate reducer: Growth of the deltaproteobacterium *Desulfurivibrio alkaliphilus* by sulfide oxidation with nitrate. mBio 8:e00671-17.
